# Editorial: The link between the Western diet and Inflammatory Bowel Disease (IBD)

**DOI:** 10.3389/fimmu.2026.1844649

**Published:** 2026-04-14

**Authors:** Shahanshah Khan, Yan Chun Li, Alex Rodriguez-Palacios

**Affiliations:** 1 Division of Digestive and Liver Diseases, Department of Internal Medicine, University of Texas Southwestern Medical Center, Dallas, TX, United States; 2 Department of Medicine, Division of Biological Sciences, The University of Chicago, Chicago, IL, United States; 3 Department of Medicine, School of Medicine, Case Western Reserve University, Cleveland, OH, United States

**Keywords:** gut micobiota, immune responce, inflammatory bowel disease, intestinal inflamamtion, western diet

Inflammatory bowel disease (IBD), which includes Crohn’s disease (CD) and ulcerative colitis (UC), is a group of chronic inflammatory disorders of the gastrointestinal tract that poses significant global health and socioeconomic burdens (1, 2). Over the past decades, both the incidence and prevalence of IBD have increased markedly, particularly in regions undergoing rapid industrialization and urbanization (3, 4). Although historically more prevalent in Western countries, a steady rise in IBD cases has been observed in developing regions, including Asia and Africa (3). This shift has drawn attention to environmental factors, particularly dietary patterns associated with the adoption of Western lifestyles, as key contributors to disease development (5).

The pathogenesis of IBD is complex and involves interactions among genetic susceptibility, environmental exposures, the gut microbiota, and immune responses (6–8). Among environmental determinants, diet has emerged as a crucial factor influencing intestinal inflammation and microbial ecology (9–11). The Western diet, characterized by high intake of saturated fats, refined sugars, processed foods, and salt, and low intake of dietary fiber and plant-derived nutrients, has been associated with several chronic inflammatory diseases (12–15). Increasing evidence suggests that this dietary pattern can alter gut microbial composition, impair epithelial barrier integrity, and modulate immune signaling pathways, thereby contributing to intestinal inflammation and disease susceptibility (9, 10, 16).

Recognizing the growing importance of nutritional factors in immune-mediated diseases, this Research Topic - *“The link between the Western diet and Inflammatory Bowel Disease (IBD)” -* was developed in the Nutritional Immunology section of *Frontiers in Immunology*. The Research Topic aims to highlight emerging research exploring how dietary components, metabolic processes, and host-microbe interactions influence the onset, progression, and systemic consequences of IBD. The studies included in this Research Topic span several complementary areas, including metabolic biomarkers of disease activity, integrative systems biology approaches to diet-disease interactions, and genetic analyses exploring broader disease associations.

One of the key themes emerging from this Research Topic is the growing recognition that metabolic alterations, particularly those related to lipid metabolism and saturated fats, are closely associated with disease activity in IBD. Chronic intestinal inflammation can influence systemic metabolic pathways, including lipid metabolism, which may serve as a useful indicator of disease status (17, 18). In a retrospective cross-sectional study, Li and colleagues examined the association between serum lipid profiles and clinical disease severity in patients with Crohn’s disease [Ni et al.]. The study found that patients with Crohn’s disease exhibited lower levels of total cholesterol (TC), high-density lipoprotein cholesterol (HDL-C), and low-density lipoprotein cholesterol (LDL-C) compared with healthy individuals. Furthermore, reduced TC levels were associated with greater disease activity and were negatively correlated with inflammatory markers and clinical disease scores. Notably, TC levels below 3.5 mmol/L were identified as an independent risk factor for increased disease severity. These findings highlight the potential utility of lipid parameters as supplementary biomarkers for assessing inflammatory activity in Crohn’s disease.

Supporting these observations, a systematic review and meta-analysis by Chen et al. examined serum lipid profiles in patients with IBD across multiple studies. By analyzing data from 53 studies, the authors reported that individuals with IBD generally display lower circulating levels of total cholesterol, HDL cholesterol, and LDL cholesterol compared with healthy controls. The analysis further revealed that lipid reductions were more pronounced in patients with active disease, suggesting a link between metabolic dysregulation and inflammatory activity. Interestingly, differences between Crohn’s disease and ulcerative colitis were also observed, indicating that lipid metabolism may be differentially affected across IBD subtypes. Together, these findings underscore the close relationship between systemic metabolism and intestinal inflammation.

Another major focus of this Research Topic involves understanding the complex biological networks through which dietary patterns influence immune responses and intestinal health. Traditional approaches often examine individual dietary components or pathways in isolation; however, the multifactorial nature of IBD requires integrative analytical strategies. In this context, Mokoena and colleagues present a comprehensive review discussing the potential of systems biology to unravel Western diet-associated triggers in IBD [Konjar et al.]. Systems biology integrates high-throughput datasets from genomics, transcriptomics, proteomics, metabolomics, and microbiome studies to build comprehensive models of biological processes. Through computational modeling and network analyses, these approaches enable researchers to identify key molecular pathways and interactions involved in disease progression. The authors emphasize that combining systems biology with nutritional epidemiology may provide valuable insights into how dietary exposures influence immune function, microbial dynamics, and metabolic regulation. Such integrative frameworks could facilitate the development of precision nutrition strategies aimed at preventing or managing IBD in populations increasingly adopting Western dietary habits.

Beyond intestinal inflammation itself, IBD is increasingly recognized as a systemic condition that may influence the risk of other diseases, including certain malignancies. Chronic inflammation, immune dysregulation, and long-term therapeutic interventions may all contribute to these associations (19, 20). In this Research Topic, Yu et al.used Mendelian randomization analysis to investigate potential causal relationships between genetically predicted IBD and the risk of extraintestinal cancers across European and East Asian populations. Their findings suggest that Crohn’s disease may be associated with increased risks of pancreatic and breast cancers in European populations, and pancreatic cancer in East Asian populations. Additionally, ulcerative colitis showed potential causal relationships with several cancers, including cervical cancer in Europeans and gastric, bile duct, hepatocellular, lung, and cervical cancers in East Asians. These results emphasize the systemic nature of immune-mediated inflammatory diseases and highlight the importance of long-term surveillance and preventive healthcare strategies for patients with IBD.

Collectively, these peer-reviewed contributions highlight the multifaceted relationship between diet, metabolism, immune regulation, and disease outcomes in IBD. Western dietary patterns may influence disease susceptibility and progression through multiple interconnected mechanisms, including alterations in lipid metabolism, disruption of microbial homeostasis, and activation of inflammatory signaling pathways. At the same time, advances in systems biology and multi-omics technologies are enabling researchers to explore these complex interactions in unprecedented detail.

Understanding how dietary factors influence intestinal inflammation also has important clinical implications. Identifying metabolic biomarkers associated with disease activity could improve disease monitoring and risk stratification. Moreover, insights into diet-microbiome-immune interactions may inform the development of targeted dietary interventions as part of integrated treatment strategies. As interest in precision medicine continues to grow, integrating nutritional science with immunology and microbiome research will likely play an increasingly important role in shaping personalized therapeutic approaches.

The continued rise in IBD incidence worldwide underscores the urgent need for multidisciplinary research that bridges nutrition science, immunology, microbiology, genetics, and computational biology. Future studies should focus on elucidating the molecular mechanisms linking dietary components to immune dysregulation, identifying key microbial and metabolic mediators of disease, and translating these discoveries into effective prevention and treatment strategies.

In summary, this Research Topic provides valuable insights into the complex interactions between Western dietary patterns and inflammatory bowel disease. By bringing together research on metabolic biomarkers, integrative systems biology approaches, and genetic epidemiology, the Research Topic contributes to a deeper understanding of how environmental and dietary factors shape intestinal immune responses. Continued exploration at the intersection of nutrition and immunology will be essential for developing innovative strategies to prevent and manage IBD in an increasingly globalized world.
